# Insights and progress on postoperative analgesia of radical gastrectomy for gastric cancer: a comprehensive review

**DOI:** 10.3389/fpain.2025.1601220

**Published:** 2025-06-30

**Authors:** Linghui Li, Yuanyuan Zhao, Huan Chen, Jianqin Zhao, Mengjun Dai, Qi Wang, Jie Lv, Wei Wang

**Affiliations:** ^1^ Department of Surgery and Anesthesiology, The Affiliated Jiangning Hospital of Nanjing Medical University, Nangjing, China; ^2^ Department of Anesthesiology, Huainan First People’s Hospital, The First Affiliated Hospital of Anhui University of Science and Technology, Huainan, China

**Keywords:** stomach neoplasms, analgesia, pain management, pain mechanisms, clinical studies as topic

## Abstract

Gastric cancer is a common and highly lethal malignancy of the digestive system, with surgical resection as the primary treatment approach. However, postoperative analgesia management remains a major clinical challenge. Postoperative pain not only affects recovery speed but may also lead to complications, thereby influencing prognosis. Recent research on postoperative pain following gastric cancer surgery has expanded, exploring various analgesic methods, including pharmacological therapy, neuraxial blocks, and non-pharmacological approaches, with growing emphasis on individualized analgesia protocols. Despite the proposal of multiple analgesic techniques, current research indicates that their effectiveness and safety are still inadequately assessed in clinical applications. This review aims to discuss the physiological mechanisms of postoperative pain following gastric cancer surgery, modern analgesic strategies, and related research, to provide a theoretical basis and clinical guidance for improving postoperative quality of life.

## Introduction

Gastric cancer is a leading cause of cancer-related mortality worldwide, with surgical resection considered the primary treatment option. Postoperative pain is a common complication in gastric cancer patients, significantly affecting recovery and quality of life, highlighting the importance of research into effective postoperative analgesia strategies (1). Recent research on postoperative analgesia following gastric cancer surgery has made notable progress, particularly in pain management techniques and pharmacological (2). However, it is still required further exploration of pain mechanisms and the development of optimal practice protocols to improve patient outcomes.

Patient-controlled intravenous analgesia (PCIA) and patient-controlled epidural analgesia (PCEA) are two primary methods for postoperative analgesia following gastric cancer surgery (3, 4). It is reported that PCEA provides superior pain relief in older adult gastric cancer patients. Compared to PCIA, patients in the PCEA group showed significantly lower Visual Analog Scale (VAS) scores on postoperative days 1, 2, and 3, along with shorter hospital stays (5). Additionally, a study indicated that in patients undergoing laparoscopic gastrectomy, PCEA demonstrated non-inferiority in pain relief compared to PCIA, with similar postoperative recovery parameters (4). These studies suggest that selecting the appropriate analgesic method is crucial for optimizing postoperative pain management. In addition to traditional analgesia methods, ultrasound-guided neuraxial blocks have been introduced into postoperative pain management for gastric cancer surgery. For example, transversus abdominis plane block (TAPB) significantly reduces opioid consumption within 24 h postoperatively and improves pain scores, particularly during coughing (6). These results suggest that TAPB, as an emerging analgesic technique, holds promise in reducing postoperative pain and opioid requirements, thereby minimizing related side effects. Furthermore, studies have shown that erector spinae plane block (ESPB) (7), rectus sheath block (RSB) (8), quadratus lumborum block (QLB) (9), paravertebral block (PVB) (10), transcutaneous electrical nerve stimulation (TEAS) (11), and intraoral acupuncture therapy (12) can reduce opioid use during the perioperative period in gastric cancer surgery patients, enhancing analgesic efficacy. The application of enhanced recovery after surgery (ERAS) protocols following gastric cancer surgery has garnered significant attention. By optimizing preoperative, intraoperative, and postoperative management, ERAS protocols can significantly reduce postoperative hospital stays and complication rates (13). Systematic reviews and meta-analyses indicate that patients undergoing ERAS protocols experience better postoperative recovery, including shorter hospital stays and lower complication rates (14). These findings offer new insights into postoperative pain management following gastric cancer surgery, emphasizing the importance of a comprehensive approach.

In this review, we discussed the physiological mechanisms of postoperative pain following gastric cancer surgery, modern analgesic strategies, and related research, aiming to provide a theoretical basis and clinical guidance for improving postoperative quality of life.

## Physiological mechanisms of postoperative pain after radical gastrectomy for gastric cancer

### Impact of surgical trauma on pain

Gastric cancer surgery typically involves extensive tissue resection and reconstruction, which can cause significant pain responses (15). The incision cuts the nerve endings in the skin, subcutaneous tissue, muscles (such as the rectus abdominis), fascia and peritoneum, which is the most direct and superficial source of pain. Moreover, gastrectomy and reconstruction, surgical traction, abdominal adhesion and changes in digestive function can lead to damage, inflammation or ischemia of organs in the abdominal cavity, which is the deeper visceral pain (16). Furthermore, the sensation of the abdominal skin is primarily supplied by specific cutaneous nerves, mainly the intercostal and subcostal nerves. These nerves run through the area where the gastric cancer surgery incision is made, particularly the upper abdominal vertical or subcostal incision in open surgery. During the operation, cutting, pulling, and suturing tissues can inevitably cause varying degrees of damage to these cutaneous nerves. Cutaneous nerve injury and neuropathic pain are the most common causes of chronic postoperative pain (17). Additionally, tissue damage activates nociceptors during surgery, leading to the release of proinflammatory mediators such as prostaglandins and interleukins. These substances not only directly stimulate nerve endings but also trigger local inflammatory reactions, which further exacerbate pain perception (18). The intensity of postoperative pain is closely related to the type of surgery, postoperative complications, and the patient's psychological state (19). Therefore, understanding the impact of surgical trauma on pain is essential for developing effective postoperative analgesia protocols.

### Role of the nervous system in pain

The nervous system plays a central role in both the perception and transmission of pain. After surgery, both peripheral and central nervous system reactions influence the intensity and duration of pain (20). Peripheral nociceptive neurons release neurotransmitters when injured or inflamed, activating spinal cord neurons that transmit pain signals to the brain (21). Furthermore, spinal cord neurons amplify pain signals during transmission, which increases pain sensitivity, a phenomenon known as “central sensitization” (22). This phenomenon is particularly evident after gastric cancer surgery, where trauma and inflammation alter nervous system function, making patients more sensitive to pain (23). It was shown that neurotransmitters and modulators in the nervous system, such as norepinephrine and serotonin, participate in pain regulation, influencing pain intensity and duration (24). Therefore, interventions targeting the nervous system, such as neuraxial blocks or pharmacological therapies, may help alleviate postoperative pain.

### Relationship between inflammatory response and chronic pain

The postoperative inflammatory response is a key mechanism in the development of chronic pain. Following surgical trauma, local tissues release a significant amount of inflammatory mediators, activating immune cells and triggering an inflammatory response (25). Although this inflammatory response is a protective mechanism, if excessive or prolonged, it may lead to chronic pain (17). It is reported that levels of inflammatory markers are often significantly elevated in patients with chronic pain, indicating a close correlation between inflammation and pain (26). Additionally, the occurrence of chronic pain is linked to plastic changes in the nervous system. Persistent inflammatory stimulation may cause structural and functional changes in neurons, creating a “memory” of pain, leading to pain persistence even without obvious stimulation (27). Psychological factors, such as anxiety and depression, are also considered to play significant roles in the development of chronic pain (28). Chronic pain not only impacts patients' quality of life but may also delay postoperative recovery. Therefore, controlling the postoperative inflammatory response and providing early psychological interventions may be crucial strategies for preventing and managing chronic pain.

### Placebo and contextual effects on postoperative analgesia

In recent years, placebo and nocebo effects have been extensively documented in different medical conditions, including pain. The placebo effect can play a prominent role in the cognitive strategies that can facilitate motor performance in sport and physical practice (29). Moreover, the invasiveness and ritual nature of the surgery may be explained through contextual effects and expectations (30). However, the study of Rossettini et al. (31) showed that there were no unique results of the occurrence and magnitude of placebo and nocebo effects in chronic pain patients, mainly due to the heterogeneity of pain.

## Application of traditional analgesia methods

### Nonsteroidal anti-inflammatory drugs (NSAIDs)

NSAIDs are commonly used for postoperative analgesia after gastric cancer surgery due to their effective analgesic and anti-inflammatory properties (32). NSAIDs alleviate pain and inflammation by inhibiting cyclooxygenase (COX), which reduces prostaglandin synthesis (33). After gastric cancer surgery, the use of NSAID can significantly reduce postoperative pain scores, decrease opioid dependence, and lower the risk of related side effects (34). However, NSAID use carries certain risks, especially in patients with gastrointestinal diseases or bleeding tendencies, as it may increase the risk of gastrointestinal bleeding and ulcers (35). It is shown that selective COX-2 inhibitors, such as meloxicam and celecoxib, offer significantly better gastrointestinal safety than traditional non-selective NSAIDs. However, their effects on cardiovascular and renal function require further investigation (36). Therefore, clinicians must weigh the analgesic benefits of NSAIDs against potential risks and select an appropriate dosing regimen.

### Use of opioids

Opioids remain a crucial option for managing postoperative pain after gastric cancer surgery, particularly in patients with severe pain (37). Common opioid receptor agonists include sufentanil, fentanyl, hydromorphone, and oxycodone, while opioid receptor agonist-antagonists include nalbuphine, butorphanol, dezocine, and the weak opioid tramadol. Opioids reduce pain perception by binding to opioid receptors in the central nervous system. However, their use carries risks of side effects, including nausea, vomiting, respiratory depression, dizziness, somnolence, and addiction (38). Research in Table 1 indicates that sufentanil and oxycodone are better suited for controlling visceral pain after gastric cancer surgery due to their high lipophilicity and strong analgesic potency. Hydromorphone's metabolism is independent of renal function, making it suitable for patients with renal insufficiency. Newer drugs, such as nalbuphine, show potential in reducing delirium and respiratory depression (39–43). In recent years, concerns about opioid abuse have led to a trend in clinical practice toward adopting multimodal analgesia protocols to reduce opioid use, thereby minimizing the incidence of related adverse reactions (44). Therefore, the rational use of opioid analgesics, combined with other analgesic methods, is key to improving the effectiveness of postoperative pain management after gastric cancer surgery.

**Table 1:** Different opioids in PCIA for gastric cancer patients.

Drug name	Recommended dosage (PCIA)	Analgesic effect (24 h VAS score)	Main side effects [incidence (%)]	Advantages/remarks	Ref/year
Sufentanil	0.02–0.04 µg/kg/h	2.8 ± 1.0	Nausea and vomiting (18%–25%); respiratory depression (3%–5%)	Potent analgesia, requires close respiratory monitoring	(38)/2022
Fentanyl	0.3–0.5 µg/kg/h	3.2 ± 1.2	Nausea and vomiting (20%–30%); pruritus (10%)	Rapid onset, significant individual variations in metabolism	(39)/2016
Hydromorphone	0.1–0.2 mg/h	2.5 ± 0.9	Dizziness (15%); drowsiness (8%)	Inactive metabolites; suitable for patients with renal insufficiency	(40)/2013
Oxycodone	0.05–0.1 mg/kg/h	2.4 ± 0.8	Nausea and vomiting (15%); constipation (10%)	Superior efficacy for visceral pain	(41)/2021
Tramadol	0.3–0.8 mg/kg/h	2.7 ± 0.6	Nausea and vomiting (30%–40%); dizziness (20%)	No respiratory depression; high incidence of nausea and vomiting	(42)/2024
Nalbuphine	0.02–0.05 mg/kg/h	3.07 ± 0.5	Nausea and vomiting (5.56%); dizziness and drowsiness (3.7%)	Comparable analgesic effect to sufentanil; reduced risk of postoperative delirium	(43)/2025

### Strategies for combined use of analgesic drugs

The combined use of analgesic drugs has shown favorable outcomes in managing pain following gastric cancer surgery (45). This strategy typically involves combining multiple analgesic drugs with different mechanisms of action, including NSAIDs, opioids, and adjuvant drugs (e.g., dexmedetomidine, ketamine, and glucocorticoids) to improve analgesic efficacy and minimize side effects. Research presented in Table 2 shows that combining PCIA with NSAIDs is economical and practical but requires assessment of gastrointestinal risks and is contraindicated in patients with coagulation disorders. By rationally selecting and combining different analgesic drugs, clinicians can develop individualized regimens tailored to each patient's specific conditions, optimizing analgesic efficacy while minimizing side effects. This strategy is increasingly recognized in modern pain management and is a crucial component of postoperative analgesia for gastric cancer.

**Table 2:** Application of non-opioid adjuvant drugs in PCIA for gastric cancer patients.

Drug name	Recommended dosage (PCIA)	Analgesic effect (24 h VAS score)	Main side effects [Incidence (%)]	Advantages/remarks	Ref/year
Flurbiprofen Axetil	1–2 mg/kg/d	2.6 ± 0.9	Gastrointestinal bleeding (2%); renal impairment (1%)	Multimodal analgesia; reduces opioid dosage; affects gastrointestinal and coagulation functions	(34)/2023
Dexmedetomidine	0.2–0.5 µg/kg/h	2.0 ± 0.6	Bradycardia (5%); hypotension (3%)	Reduces opioid consumption; improves sedation scores; requires heart rate monitoring	(46)/2024
Ketamine	0.1–0.3 mg/kg/h	2.3 ± 0.7	Hallucinations (5%); excessive dreaming (3%)	Inhibits central sensitization; reduces postoperative hyperalgesia; requires vigilance against neuropsychiatric side effects	(47)/2016
Dexamethasone	0.2–0.4 mg/h	3.0 ± 1.2	Nausea and vomiting (25%); pruritus (15%); somnolence (10%)	Reduces opioid dosage; requires attention to immunosuppression risks	(48)/2024

## Emerging analgesic techniques

### Application of peripheral nerve blocks

Peripheral nerve blocks, particularly ultrasound-guided techniques, have gained increasing attention for postoperative analgesia in gastric cancer patients (49). Research in Table 3 shows that TAPB, ESPB, RSB, QLB, and PVB provide significant analgesic effects after gastric cancer surgery, reducing opioid consumption and postoperative pain scores within 24 h. TAPB, ESPB, and QLB offer advantages in laparoscopic surgery by reducing opioid dependence. Combined blocks (e.g., TAPB + ESPB) may improve visceral pain control, although further clinical evidence is needed to support this (6–10). These findings suggest that nerve block techniques may have opioid-sparing effects in postoperative analgesia, thereby reducing opioid-related adverse reactions.

**Table 3:** Applications of different nerve block techniques in postoperative analgesia for gastric cancer patients.

Nerve block technique	Technique description	Analgesic effect	Complications	Applicability	Ref/year
Transversus abdominis plane block (TAPB)	Local anesthetic is injected into the transversus abdominis plane (between the internal oblique and transversus abdominis muscles) to block the nerves innervating the abdominal wall and skin	Reduces VAS scores at 48 h postoperatively and decreases perioperative opioid use.	Local anesthetic toxicity; puncture injury	Abdominal and abdominal wall surgeries	(6)/2022
Erector spinae plane block (ESPB)	Local anesthetic is injected under ultrasound guidance into the deep surface of the erector spinae muscle to block the posterior branches of the thoracic spinal nerves	Provides coverage of thoracic spinal nerves, reducing VAS scores by 40%–50% at 48 h postoperatively.	Rare respiratory depression; local anesthetic spread into the spinal canal	Open or laparoscopic radical gastrectomy, especially for T_6_–T_9_ segmental blocks.	(7)/2023
Rectus sheath block (RSB)	Local anesthetic is injected between the rectus abdominis muscle and its posterior sheath to block the anterior cutaneous branches of the intercostal nerves	Reduces VAS scores at 48 h postoperatively and decreases perioperative opioid use.	Abdominal organ injury; local anesthetic toxicity	Analgesia for surgeries through the abdominal midline or rectus abdominis incision.	(8)/2023
Quadratus lumborum block (QLB)	Local anesthetic is injected under ultrasound guidance around the quadratus lumborum muscle to block the thoracolumbar nerves	Provides analgesic effects comparable to TAPB at 24 h postoperatively, with slightly better visceral pain control.	May cause transient lower limb weakness.	Laparoscopic gastric cancer surgery, with attention to anatomical variations.	(9)/2022
Paravertebral block (PVB)	Local anesthetic is injected into the paravertebral space to block the spinal nerve, their branches, and sympathetic nerves at that segment.	Simultaneously inhibits somatic and visceral pain, reducing VAS scores at 48 h postoperatively and decreasing postoperative opioid use.	Puncture of the pleura or lungs, nerve root injury; high-level block	Upper abdominal and chest surgeries.	(10)/2024

### Application of central analgesia techniques

Central analgesia techniques, such as PCEA, are crucial in managing postoperative pain following gastric cancer surgery. Studies have shown that PCEA provides superior analgesia and results in better postoperative recovery compared to PCIA (3, 4). A comparative study showed that patients undergoing laparoscopic gastrectomy who received epidural analgesia had significantly lower pain scores within 24 h postoperatively and shorter hospital stays than those in the PCIA group. However, the epidural group had higher risks of hypotension, urinary retention, and motor blockade, requiring hemodynamic monitoring during the perioperative period (4). Epidural analgesia also accelerates gastrointestinal function recovery, which is crucial for the postoperative rehabilitation of gastric cancer patients (50). Additionally, it is reported that a single intrathecal morphine injection for analgesia in patients after radical gastrectomy can alleviate postoperative pain. However, the duration of analgesia is significantly shorter, and its controllability is inferior to that of PCEA (51). These findings suggest that central analgesia techniques are valuable in improving postoperative pain management and facilitating recovery.

### Impact of physical therapy on postoperative pain

Physical therapy has demonstrated positive effects in managing pain following gastric cancer surgery. Research indicates that multimodal physical therapy interventions, when implemented postoperatively, can significantly improve pain perception and functional recovery in patients. For example, studies using TEAS or acupuncture therapy as adjuvant therapy have shown that TEAS effectively reduces pain after gastric surgery, decreases perioperative opioid use, and improves digestive function (11, 52, 53). These results suggest that physical therapy, as part of a comprehensive treatment plan, can effectively alleviate postoperative pain and promote recovery, supporting its widespread application in pain management following gastric cancer surgery.

## Impact of psychological factors on postoperative pain management

Psychological factors play a vital role in postoperative pain management (54). It is shown that patients' psychological states, especially anxiety and depression, significantly influence pain perception and the effectiveness of pain management (55). Anxiety and depression not only exacerbate patients' subjective pain experience but also increase the demand for analgesic medications, impacting postoperative recovery and quality of life (56). Therefore, interventions targeting psychological factors are essential in postoperative pain management.

### Relationship between anxiety and pain perception

Anxiety is a significant psychological factor influencing postoperative pain perception (57). It is reported that anxiety influences pain perception by increasing sensitivity to pain and decreasing tolerance, with patients who report higher preoperative anxiety levels often experiencing more severe postoperative pain (24). Specifically, anxious patients may have stronger emotional responses to pain, intensifying their pain experience (58). Furthermore, anxiety increases pain expectancy, which in turn exacerbates pain perception (59). Therefore, assessing and managing preoperative anxiety is vital for enhancing postoperative pain management.

### Effectiveness of psychological intervention measures

Psychological interventions have shown positive effects in improving postoperative pain management (60). Psychological interventions, such as cognitive-behavioral therapy (CBT) and relaxation training, can effectively reduce anxiety levels, thereby alleviating postoperative pain (61). For example, a study found that patients who received psychological interventions had significantly lower postoperative pain scores than those who did not, and reduced their dependence on medications, thereby lowering potential drug side effects (62). Additionally, psychological interventions improve patients' psychological states, enhance their coping abilities, and promote overall postoperative recovery outcomes (63). Therefore, incorporating psychological interventions into postoperative pain management can significantly improve patient satisfaction and quality of life.

### Patient education and self-management

Patient education is equally essential in postoperative pain management (64). By providing knowledge and skills for pain management, patients can better understand and cope with postoperative pain, thereby improving their self-management abilities (65). Moreover, educating patients on how to use analgesic medications, recognize pain signals, and adopt effective self-management strategies can significantly reduce postoperative pain perception (66). Furthermore, patient education helps establish reasonable expectations and reduces fear of pain, thereby improving psychological well-being and postoperative recovery outcomes (64). In summary, integrating psychological interventions, patient education, and self-management strategies will enhance the overall effectiveness of postoperative pain management.

## Application of alternative therapies

Alternative therapies, including traditional Chinese medicine, massage, and acupuncture, are gaining attention in the management of postoperative pain following gastric cancer surgery (67). These therapies target pain management through various mechanisms, effectively alleviating postoperative pain and promoting overall rehabilitation. For example, it is shown that traditional Chinese nursing interventions significantly reduce postoperative pain scores and improve patients' physiological and psychological states (68). Buccal acupuncture therapy, which stimulates various acupuncture points, can enhance the effectiveness of PCIA in patients undergoing laparoscopic radical gastrectomy for gastric cancer, reduce opioid use, and facilitate postoperative recovery (12). Additionally, alternative therapies generally have fewer side effects and are suitable for long-term use, offering patients more treatment options (69). With further research into their effectiveness and safety, alternative therapies are expected to play an increasingly significant role in pain management in the future.

## Multimodal analgesia strategies

Multimodal analgesia is the established gold standard for postoperative pain management in gastric cancer patients. It aims to achieve synergistic analgesic effects while reducing the dosage and adverse effects of single-agent therapies, particularly opioids, through the combination of pharmacologically distinct agents and techniques (70, 71). The formulation of an analgesic regimen should account for surgical approach, patient-specific factors, and ERAS protocols. For example: PCEA combined with NSAID) and gabapentin is recommended in open gastrectomy (3), while TAPB or ESPB with a selective COX-2 inhibitor is advised in Laparoscopic gastrectomy (7, 8). For older adult or renally impaired patients, NSAIDs are contraindicated and regional nerve blocks are preferred (36). Moreover, Adjunctive ketamine may be utilized for opioid-tolerant patients (47). Within ERAS pathways, the key objectives include maintaining VAS scores <4 within 24 h postoperatively, minimizing opioid consumption, and discontinuing opioids within 48 h when feasible (72). The current consensus advocates a four-step analgesic protocol: priority regional anesthesia (PCEA/TAP/ESPB), Maximized non-opioid foundation (acetaminophen + COX-2 inhibitor), opioids as rescue therapy (preferably via PCIA) and dynamic reassessment at 72 h (regimen adjustment until VAS ≤4) (70, 71).

## Future research directions and challenges

### Exploration of individualized analgesic strategies

The exploration of individualized analgesic strategies is crucial in postoperative pain management following gastric cancer surgery. Research has shown that personalized analgesic regimens can significantly improve postoperative recovery and quality of life (73). By analyzing patients' physiological characteristics, psychological states, and pain perceptions, medical teams can create more tailored analgesic plans. Individualizing analgesic approaches based on these individual differences enhances analgesic effectiveness and reduces the risk of adverse drug reactions. For example, using biomarkers to predict the severity of postoperative pain can help anesthesiologists select the most appropriate analgesic medications and administration methods, achieving individualized pain management (74). Advances in genomics and pharmacogenomics allow researchers to predict patients' responses to specific analgesics, optimizing medication selection (75). Additionally, patients' personal pain goals (PPG) should be considered to ensure pain management plans align with their needs and expectations (76). Therefore, future research should focus on developing more precise assessment tools to tailor individualized analgesic strategies to patients' specific needs early in the postoperative period.

### Importance of multidisciplinary collaboration in pain management

Multidisciplinary collaboration is crucial in pain management, especially in complex cases like those involving gastric cancer surgery patients. By integrating the expertise of surgeons, anesthesiologists, pain management specialists, and nursing teams, a more comprehensive pain management plan can be developed (77). For example, anesthesiologists can provide targeted analgesic techniques, while surgeons can adjust analgesic strategies based on surgery type and patient condition. Research shows that interdisciplinary collaboration improves pain control outcomes and reduces postoperative complications (78). Additionally, establishing effective communication mechanisms and collaboration platforms is key to enabling multidisciplinary cooperation (79). Future research should explore ways to optimize multidisciplinary team collaboration models to enhance pain management effectiveness following gastric cancer surgery and improve patients' quality of life.

### Development of novel analgesic drugs

As understanding of pain mechanisms deepens, the development of novel analgesic drugs has become a key focus for improving postoperative pain management following gastric cancer surgery (80). Currently, traditional analgesics, such as opioids, present a challenge in balancing efficacy with side effects (81). Novel drugs, including neuropeptide-based medications and targeted therapies, are being extensively studied. These drugs target pain pathways through various mechanisms, offering more effective pain relief with fewer side effects (82). Additionally, nano-drug delivery systems offer new possibilities for analgesic drug development by enabling more precise drug release and minimizing side effects (83). Moreover, drug discovery methods that leverage artificial intelligence and big data technologies are accelerating the development of analgesic drugs (84). Future research should focus on the clinical application of these novel drugs, evaluating their effectiveness in pain management following gastric cancer surgery, and exploring their potential for personalized treatments to provide safer and more effective analgesic solutions for patients.

## Limitations

There were several limitations in this review. First of all, this paper mainly reviewed the postoperative analgesic regimen for patients with radical gastrectomy, and did not conduct a specific meta-analysis. Secondly, perhaps some pictures could improve the quality of this review, but the tables in the manuscript have covered the main analgesic drugs and protocols currently being focused on and applied. Additionally, as the mainstream method of regional anesthesia techniques, peripheral nerve block were more extensive and effective than local infiltration anesthesia, which was not discussed in this review.

## Conclusions

Existing studies suggest that traditional pharmacological treatments remain the primary approach for alleviating postoperative pain in gastric cancer patients. However, as understanding of pain mechanisms deepens, emerging techniques such as neural blockade, ultrasound-guided analgesia, and multimodal analgesic strategies are increasingly showing their importance. Despite the abundance of literature on different analgesic techniques and their effects, balancing the findings from various studies remains a challenge in practical applications. Therefore, future research should emphasize developing personalized pain management plans based on patients’ specific conditions. Integrating psychological interventions with pain management will enhance patients' quality of life and postoperative recovery outcomes, an area ripe for further exploration and validation. In summary, the prospects for research in postoperative pain management following gastric cancer surgery are broad. Future research should emphasize individualized and comprehensive management strategies to meet the needs of diverse patients, ultimately enhancing postoperative quality of life and satisfaction.
